# Micro-epidemiology of *Plasmodium falciparum *malaria: Is there any difference in transmission risk between neighbouring villages?

**DOI:** 10.1186/1475-2875-6-46

**Published:** 2007-04-19

**Authors:** Yazoumé Yé, Catherine Kyobutungi, Valérie R Louis, Rainer Sauerborn

**Affiliations:** 1 African Population and Health Research Centre, PO Box 10787-00100 GPO Nairobi, Kenya; 2 Department of Tropical Hygiene and Public Health, University of Heidelberg, Medical School, Heidelberg, Germany

## Abstract

**Background:**

Malaria control strategies are designed as a solution for either the whole region or the whole country and are assumed to suit every setting. There is a need to shift from this assumption because transmission may be different from one local setting to another. The aim of this study was to assess the risk of clinical malaria given the village of residence among under-five children in rural north-western Burkina Faso.

**Methods:**

867 children (6–59 months) were randomly selected from four sites. Interviewers visited the children weekly at home over a one-year period and tested them for fever. Children with fever were tested for malaria parasites. An episode of clinical malaria was defined as fever (axillary temperature ≥ 37.5°C) + parasites density ≥ 5,000 parasites/μl. Logistic regression was used to assess the risk of clinical malaria among children at a given site of residence.

**Results:**

Children accumulated 758 person years (PYs). Overall, 597 episodes of clinical malaria were observed, giving an incidence rate of 0.79 per PY. The risk of clinical malaria varied amongst the four sites. Taking one village as reference the odds ratio for the other three sites ranged from 0.66; 95%CI: 0.44–0.98 to 1.49; 95%CI: 1.10–2.01.

**Conclusion:**

Malaria control strategies should be designed to fit the local context. The heterogeneity of transmission should be assessed at the district level to allow cost-effective resource allocation that gives priority to locations with high risk. Functional routine health information systems could provide the necessary data for context specific risk assessment.

## Background

Malaria remains a public health concern worldwide and especially in sub-Saharan Africa (SSA), where it is a major killer among children under five years of age [1-4]. This is despite local, national, and international efforts to control the disease. A country or a region in the country may be classified as endemic or holo-endemic or meso-endemic depending on the transmission intensity [5]. Subsequently, control strategies are designed for the whole region or country and are assumed to suit every local setting therein. However, the transmission may be different from one local setting to another within the same region or geographical locality and interventions designed at the regional or national level may fail to meet needs on the ground. It may be assumed that health districts would focus resources for malaria control proportionate to risk of malaria transmission in specific localities if they were aware of which localities were more threatened than others. Knowledge of malaria risk at a local level may prove more informative for formulating control strategies by supporting resource allocation decisions at the health district level.

There is evidence that malaria transmission risk varies even on the smallest scale. Wang et al have demonstrated inter-city variation of malaria prevalence among school children [6]. In Dar es Salaam, malaria prevalence was shown to gradually increase as one moved from the city centre to the periphery. Similar findings were observed in Ouagadougou and Abidjan [6,7]. These studies were, however, cross-sectional in nature and were limited to urban areas. Some studies have shown that the transmission risk as measured by the entomological inoculation rate (EIR) may differ between neighbouring localities, but few studies have been carried out on the risk in terms of outcomes in the human host. The aim of this study was, therefore, to assess whether there were differences in the risk of clinical malaria (defined as axillary temperature ≥ 37.5°C and parasites density ≥ 5,000 parasites/μl) among children under-five in one town and three villages in north-western Burkina Faso.

## Methods

### Study sites

The study was conducted in the town of Nouna and the villages of Cissé, Goni, and Kodougou. These four sites are part of the Nouna Demographic Surveillance System (DSS) area [8], which is located in Kossi province in the north western part of Burkina Faso [9]. Kossi province (area 5,000 sq km) is considered a holo-endemic area. The villages are about 19–44 kilometres from each other's centre.

### Study population

A cohort of 867 children (Cissé: 171, Goni: 240, Kodougou: 191 and Nouna: 265) aged 6 to 59 months, participated in the study. The children were selected from each site by means of cluster sampling of households using a sampling frame generated from the DSS database. The sample size was set to detect inter-site differences in the incidence of clinical malaria of at least 10% (if they exist), with 80% power and 95% confidence, and in anticipation of 15% loss to follow up. The Local Ethics Committee of Nouna approved the study.

### Clinical malaria data

Four trained-interviewers, one based in each site, visited individual children at home every week from December 2003 to November 2004. This follow up period of 12 months covered one dry and one rainy season. At each visit, the interviewers measured the axillary temperature of the child and collected a blood sample (by finger prick) from any child with fever. In addition, they asked the mother/caretaker about the use of mosquito nets and collected data on housing conditions including the presence of animals, and potential breeding sites within a 30-metre radius of the house. This information was cross-checked by observation.

A field supervisor regularly collected the forms and blood slides from the field and transported them to the Nouna Health Research Centre laboratory. Two laboratory technicians read the blood slides stained with Giemsa to determine the parasite count. In cases of non-agreement, a biologist read the slide. This happened with only 2% of the slides. Parasite density was estimated by counting parasites in 100 fields and equating the found value to 0.25 μl. The outcome of interest was clinical malaria episode, defined as axillary temperature ≥ 37.5°C plus a parasite density ≥ 5,000 parasites/μl. This case definition is similar to the one used by previous studies in this region [10,11].

For ethical reasons, interviewers treated every child with fever for malaria, in accordance with national treatment guidelines. When fever persisted for two days or other symptoms surfaced, they referred the child to the nearest health facility. The project covered all related costs.

### Data management and quality control

Data entry clerks entered the data into a relational database developed in Microsoft Access. The data entry screen was similar to the data collection forms and included automatic consistency checks. At the field level, supervisors visited the interviewers and checked the filled forms before sending them for data entry. At the data entry level, a data entry supervisor randomly checked 5% of data entered each day. The critical proportion of error was set to the 5% level. Data was to be re-entered completely if the proportion of found errors was above 5%. However, this situation did not occur. Clean data were exported to STATA 8.1 for statistical analysis.

### Statistical modelling

Logistic regression was used to estimate the odds ratio (OR) of clinical malaria associated with residing in one of the four sites using the Goni site (site with the most episodes of clinical malaria) as a reference. The multivariate model included a number of potential confounders and these are shown in Table 1.

**Table 1:** List and description of variables included in the logistic regression model

**Factors**	**Variable**	**Description**	**Type**	**Value**
**Outcome**	*Clinical malaria episode*	Fever + Parasites density ≥ 5000 parasites/μl	Binary	0 = No, 1 = Yes
**Explanatory**				
	Site	Site of residence of the child	Categorical	1 = Nouna 2 = Cissé, 3 = Goni (ref) 4 = Kodougou
**Co-variables**				
	Gender	Sex of the child	Binary	0 = Male (ref), 1 = Female
	Age	Age group in months	Categorical	0 = < 12 1 = 12–23, 2 = 24–35 3 = 36–47, 4 = 48+(ref)
	Ethnicity	Ethnic group to which the child belongs	Categorical	1 = Peulh (ref), 2 = Mossi, 3 = Marka, 4 = Bwaba, 5 = Samo, 6 = other
	Net use	Did the child sleep under a mosquito net the since the last visit?	Binary	0 = No (ref), 1 = Yes
	Water point	Presence of a well within 30-metre radius of the house	Binary	0 = No (ref), 1 = Yes
	Farm	Presence of a farm within 30- metre radius of the household	Binary	0 = No (ref), 1 = Yes
	Animal enclosure	Presence of an animal enclosure within 30-metre radius of the house	Binary	0 = No (ref), 1 = Yes
	Breeding site	Presence of an open water body within 30-metre radius of the house	Binary	0 = No (ref), 1 = Yes
	Season	Dry season = November to May, Rainy season = June to September	Binary	0 = Dry season 1 = Rainy season

ref = reference categories

The model was built as follows:

log*it*(*π*) = *β*_0 _+ *β*_1_*Site_Cisse*_*i *_+ *β*_2_*Site_Nouna*_*i *_+ *β*_3_*Site_Kodougou*_*i *_+ *β*_4_*Gender_Female*_*i *_+ *β*_5_*Age*_ < 12_*i *_+ *β*_6_*Age*_12 - 23_*i *_+ *β*_7_*Age*_24 - 35_*i *_+ *β*_8_*Age*_36 - 47_*i *_+ *β*_9_*Ethnic_Mossi*_*i *_+ *β*_10_*Ethnic_Marka*_*i *_+ *β*_11_*Ethnic_Bwaba*_*i *_+ *β*_12_*Ethnic_Samo*_*i *_+ *β*_13_*Ethnic_other*_*i *_+ *β*_14_*Netused_Yes*_*i *_+ *β*_15_*Waterpoint_Yes*_*i *_+ *β*_15_*Farm_Yes*_*i *_+ *β*_16_*Animalenclosure_Yes*_*i *_+ *β*_17_*Breedingsite_Yes*_*i *_+ *β*_18_*Season_Rainy*_*i*_

Where *π*_*i *_is the predicted probability of having clinical malaria of the *i*th child; the odds of the same child will be πi1−πi
 MathType@MTEF@5@5@+=feaafiart1ev1aaatCvAUfKttLearuWrP9MDH5MBPbIqV92AaeXatLxBI9gBaebbnrfifHhDYfgasaacH8akY=wiFfYdH8Gipec8Eeeu0xXdbba9frFj0=OqFfea0dXdd9vqai=hGuQ8kuc9pgc9s8qqaq=dirpe0xb9q8qiLsFr0=vr0=vr0dc8meaabaqaciaacaGaaeqabaqabeGadaaakeaadaWcaaqaaGGaciab=b8aWnaaBaaaleaacqWGPbqAaeqaaaGcbaGaeGymaeJaeyOeI0Iae8hWda3aaSbaaSqaaiabdMgaPbqabaaaaaaa@352D@. *β*_0 _is the intercept and *β*_1 _... *β*_17 _the regression coefficients of the independent variables (name following each coefficient). The odds ratio associated with a given coefficient is the exponential of the respective beta coefficient. *Site_Cissé *compared to Goni (reference) is the exponential of *β*_1 _(OR_*Site_Cissé *_= exp (*β*_1_)).

## Results

### Study population characteristics

The 867 children were from 427 households and the average number of children per household was two. Kodougou had the largest mean number of children per household (2.5). Overall, females were more (52.5%) than males but the sex distribution across the sites was not significantly different (*p value *= 0.420). Similarly, the age distribution did not differ between sites (*p value *= 0.938). Participants were almost equally distributed between the age groups except for the age group below 12 months (9.2%) (Table 2)

**Table 2:** Descriptive characteristics of the study population in four sites of the Nouna DSA, Burkina Faso, from 01.12.2003 to 30.11.2004

		**Sites**				
			
	**All (%)**	**Cissé (%)**	**Goni (%)**	**Kodougou (%)**	**Nouna (%)**	*x^2 ^***test***
**N**	**867**	**171**	**240**	**191**	**265**	**-**
**Household**	427	74	125	77	151	
Children/household**	2.0	2.3	1.9	2.5	1.8	
***Gender***						*p value *= 0.420
Female	455 (52.5)	103 (60.2)	116 (48.3)	98 (51.3)	138 (52.1)	
Male	412 (47.5)	68 (39.8)	124 (51.7)	93 (48.7)	127 (47.9)	
**Age in months**						*p value *= 0.938
< 12	80 (9.2)	14 (8.2)	19 (7.9)	15 (7.9)	32 (12.1)	
12–23	200 (23.1)	39 (22.8)	57 (23.8)	43 (22.5)	61 (23.0)	
24–35	202 (23.3)	36 (21.1)	59 (24.6)	43 (22.5)	64 (24.2)	
36–47	211 (24.3)	40 (23.4)	58 (24.2)	50 (26.2)	63 (23.8)	
48+	174 (20.1)	42 (24.6)	47 (19.6)	40 (20.9)	45 (17.0)	

*Mantel-Haenszel chi-Square test for a difference across sites, **Average number of children per household

### Follow up status

**Figure 1:**
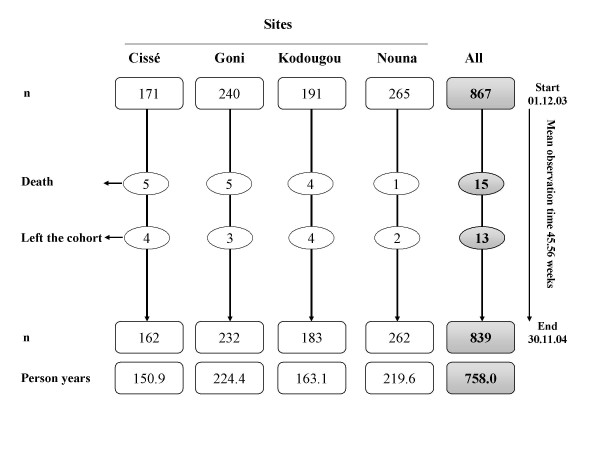
Outcome of the follow up of study participants in four sites of the Nouna DSA, Burkina Faso, from 01.12.2003 to 30.11.2004.

During the one-year observation period, 28 children left the cohort, either due to death (15) or due to migration out of the study sites (13) (Figure 1). Although fifty two home visits were planned per child, on average each child was observed for 45.6 weeks because children were not always present at each visit. As a result, the person time observed is different from the number of children. Overall, there were 758.0 observation person-years (PYs). The PYs per site were for Nouna (219.6), Kodougou (163.1) Goni (224.4), and Cissé (150.9).

### Clinical malaria incidence

The total number of observed episodes of fever was 1,635, giving an incidence of 2.2 episodes per PY (1,635 episodes/758.0 PYs). This incidence was similar in all sites (Cissé = 2.1, Goni = 2.3, Kodougou = 2.2, and Nouna = 2.0). Out of the 1,635 fever episodes, 597 were confirmed as being due to clinical malaria, giving an incidence rate of 787.6 per 1,000 PYs. The highest incidence rate was observed in Goni (935.8 per 1,000 PYs), followed by Kodougou (821.6 per 1,000 PYs), Cissé (775.3 per 1,000 PYs) and Nouna (751.4 per 1,000 PYs).

### Site of residence as a risk factor for clinical malaria

The results of the logistic regression (Table 3) showed that compared to Goni, children living in Nouna and Cissé (OR = 0.70; 95%CI: 0.52–0.95 and OR = 0.66; 95% CI: 0.44–0.98 respectively, had a significantly lower risk of clinical malaria, but those residing in Kodougou had significantly higher risk (OR = 1.49; 95%CI 1.10–2.01). Among other covariates, age, "presence of farm", "ethnicity" and "rainy season" had significant effects on the clinical malaria risk. The risk of clinical malaria decreased significantly with increasing age. The risk was 2.3 times and 1.6 times higher in children below 12 months and those between 36–47 months respectively compared to those aged 48+ months. Children of Mossi and Samo ethnicity had a significantly lower risk of malaria infection compared to Peulh (OR = 0.56; 95%CI: 0.37–0.85 and OR 0.51; 95%CI: 0.31–0.85 respectively). Children of other ethnicities had a lower but not statistically significant risk. Farming activity within a 30 metre-radius of the house was associated with significantly higher odds of clinical malaria (OR = 1.46; 95%CI: 1.21–1.78). Children were more likely to have clinical malaria during the rainy season compared to the dry one (OR = 2.19; 95%CI: 1.82–2.65).

**Table 3:** Odds ratios of clinical malaria infection among children given their site of residence

**Variables**		**PYs**	**Episodes**	**OR**	***p-value***	**95% CI**
**All**		**758.0**	**597**			
**Explanatory**						
**Site**	Goni	224.4	210	1		
	Nouna	219.6	165	0.70*	0.023	0.52–0.95
	Cissé	150.9	117	0.66*	0.042	0.44–0.98
	Kodougou	163.1	134	1.49**	0.009	1.10–2.01
**Co-variables**						
**Gender**	Male	367.6	280	1		
	Female	390.4	317	1.01	0.889	0.86–1.19
**Age in months**	> 12	14.7	10	2.28*	0.015	1.18–4.42
	12–23	149.3	144	2.09***	0.000	1.63–2.67
	24–35	168.4	164	1.98***	0.000	1.10–2.02
	36–47	178.7	145	1.60***	0.000	1.25–2.04
	48+	246.8	134	1		
**Ethnicity**	Peulh	151.5	123	1		
	Mossi	219.2	176	0.56**	0.007	0.37–0.85
	Marka	247.7	206	0.81	0.280	0.55–1.19
	Bwaba	59.6	53	0.74	0.238	0.45–1.22
	Samo	69.3	33	0.51**	0.009	0.31–0.85
	Others	10.7	6	0.80	0.611	0.33–1.92
**Net use**	No	456.3	229	1		
	Yes	301.7	368	1.14	0.186	0.94–1.40
**Water point**	No	653.7	526	1		
	Yes	104.3	71	0.91	0.574	0.65–1.27
**Farm**	No	476.7	271	1		
	Yes	281.3	326	1.46***	0.000	1.21–1.78
**Animal enclosure**	No	266.9	227	1		
	Yes	491.1	370	0.95	0.612	0.79–1.15
**Breeding site**	No	697.3	548	1		
	Yes	60.7	49	1.02	0.921	0.69–1.50
**Season**	Dry	443.5	225	1		
	Rainy	314.5	372	2.19***	0.000	1.82–2.65

* Significant at the 0.05 level, ** Significant at the 0.01 level, *** Significant at the 0.001 level, PYs = Person years, OR = Odds ratio

## Discussion

There were differences in the risk for clinical malaria between the four sites as shown by the number of episodes per PY and the odds of clinical malaria given the site of residence. The largest incidence rate was observed in Goni, which had about 1.2 times higher incidence than Nouna and Cissé. The odds ratio of clinical malaria for this site was also higher even though the highest odds ratio was found in Koudougou. Such differences were observable despite the relative proximity of the four sites since they are within a 19–44 kilometre radius of each other.

The lower incidence observed in Nouna and Cissé and Kodougou compared to Goni could be explained by differences in the ecological settings of these sites. Goni is located in a plain where there is seasonal rice farming which creates favourable habitats for mosquitoes. Kodougou is a village about 200 metres from a permanent river, which serves as a water source for traditional irrigation. Proximity to irrigated and flooded agriculture practices have been reported to be highly associated with malaria infection [12-14]. The semi-urban setting of Nouna could explain its comparatively lower incidence of clinical malaria. Indeed, urban settings are reported to be less prone to malaria transmission compared to rural settings [15] due to the pollution of water bodies therein. These polluted waters are unsuitable as breeding sites for the malaria vector. The findings of low incidence of clinical malaria from Cissé are surprising. Cissé is located next to a forest and is surrounded by a swamp during the rainy season. These conditions are suitable for an increased risk of malaria transmission. A possible explanation of the low observed risk could be presence of cattle, which serve as an alternative host of the malaria vector. Cattle keepers with their attendant animal enclosures within the villages are the main inhabitants of Cissé. An investigation of the feeding preference of malaria vectors is needed to determine the fraction of vectors feeding on animals.

Regarding the results from the logistic regression, the pattern observed in the crude incidence rate of clinical episodes per 1000 PYs was maintained after accounting for variables that may influence this distribution. Compared to children living in Goni, those living in Kodougou were more likely to have clinical malaria while those from Cissé and Nouna were significantly less likely to have it. Differences in the ecological settings of the sites as discussed above could still explain these differences in risk. Other covariates showed different effects on the risk of contracting clinical malaria.

Older children were better protected than the young ones. The odds were much higher in the age group below 12 months. This was not surprising because children below 6 months have immunity acquired from the mothers, which they progressively loose, as they grow older. Reyburn *et al *[16] reported a high risk of severe malaria in the age group below 12 months. With increasing age, children develop partial immunity and become less susceptible, although below five years this immunity remains weak.

Studies have suggested that ethnicity has a significant effect on the clinical malaria risk. Studies in this area have shown that the Peuhl (also known as Fulani) are less susceptible to malaria due to their reduced genetic susceptibility compared to other ethnicities [17,18]. However, our findings did not confirm this. Mossi and Samo have significantly lower risk than the Peuhl. Ethnic differences observed in previous studies may be attributable to differences in local ecological settings since people of the same ethnic group tend to aggregate in the same village. Previous studies did not assess this latter factor. Studies specifically designed to explore this association in this region may be of interest, as understanding genetic susceptibility to clinical malaria could prove useful for vaccine development.

Self-reported usage of a mosquito net was surprisingly not associated with a decrease in clinical malaria risk among children as has been extensively reported in other studies [19-24]. The question was precise enough to get the required information because interviewers asked mothers at each visit whether the child had been sleeping under a mosquito net since the previous visit. Although a distinction was made between normal mosquito nets and insecticide-treated nets (ITN) during data collection, this was not taken into consideration in the analysis. This was because of the very small number of non-impregnated mosquito nets, as an ongoing community trial provided ITN freely.

Among the factors related to the housing conditions, only the presence of farming activities within a 30-metre radius of the household was associated with increased risk of clinical malaria. The agriculture practice in this region is to dig small furrows between the crops to retain rainfall water for better ground infiltration. Such water bodies combined with the shadow provided by the crops create a suitable environment for vector breeding, thereby increasing the risk of clinical malaria.

The rainy season, as expected is associated with an increase of clinical malaria episodes. In the region where the climate is mostly dry, rainfall, occurring during a specific period of the year, is the main source of breeding sites (open water bodies) for the malaria vector. In contrast, during the dry season, the hot and dry weather is unsuitable for vectors, thereby leading to high vector mortality and reduction in infection. These findings are consistent with those from other studies [25,26]

The implication of these findings is that malaria control strategies should be designed to fit location-specific contexts. The distribution of the factors responsible for malaria transmission may lead to different malaria transmission patterns even within such a relatively small geographical entity. At the level of a health district or even for national malaria control policies, it would be very informative to know the risk difference between locations as small as a village. Scarce resources for malaria control could then be allocated accordingly. While all villages at risk should receive malaria control measures, high-risk villages can be identified and given priority.

Conducting such studies at the village level in every health district is not only time consuming, but it is costly. Additionally there are very few such studies already available for use. It is, therefore, imperative that well functioning health information systems are established and utilized. In practice, this will require health districts to collect routine health information data series (for example over a three-year period) to assess which villages or localities are at the highest risk, accounting for seasonal variation. This risk assessment may then be utilized to decide to which localities malaria control efforts should be focused rather than distributing resources equally or otherwise based on other criteria.

## Authors' contributions

YY designed and coordinated the implementation of the study. He performed the statistical analysis and drafted the manuscript. CK and VL participated in statistical analysis, writing of the manuscript and interpretation of the findings. RS participated in the design and implementation of the study, and writing of the manuscript. All authors read and approved the final manuscript.

## Conflict of interest

The author(s) declare that they have no competing interests.
